# Consumer and Market Antecedents of Repurchase Intention: Fear of Missing Out and Impulsive Buying as Serial Mediators

**DOI:** 10.3390/bs16060871

**Published:** 2026-05-31

**Authors:** Yang Du, Kui Deng, Ziyang Liu

**Affiliations:** Department of Global Business Graduate School, Kyonggi University, Suwon 16227, Republic of Korea; duyang@kyonggi.ac.kr (Y.D.); dengkui123@kyonggi.ac.kr (K.D.)

**Keywords:** Fear of Missing Out (FoMO), Impulsive Buying, Repurchase Intention, scarcity, cultural and creative products, Social Comparison Theory, Self-Determination Theory

## Abstract

Fear of Missing Out (FoMO) has become a salient emotion in consumer markets shaped by social media, scarcity appeals, and social display. Yet limited research has examined FoMO as a consumption-specific emotion associated with consumer dispositions, situational cues, and post-purchase intentions. Drawing on Social Comparison Theory, Self-Determination Theory, and the Stimulus–Organism–Response (S-O-R) framework, this study examines the relationships among Materialism, Envy, Scarcity, FoMO, Impulsive Buying (IB), and Repurchase Intention (RI). Survey data from 518 Chinese consumers with prior Pop Mart purchasing experience were analyzed using PLS-SEM. The results show that Materialism, Envy, and Scarcity were positively associated with FoMO, with Scarcity showing the strongest relationship. FoMO was positively associated with IB, and IB was positively associated with RI. The results also supported three serial mediation paths, indicating that FoMO and IB served as sequential mediators between Materialism, Envy, Scarcity, and RI. This study extends FoMO research to cultural and creative product consumption and provides insight into how consumption-related emotions are associated with sustained purchase tendencies.

## 1. Introduction

As social media has become embedded in everyday life, the ways consumers obtain product information, perceive market trends, and observe others’ consumption choices have changed substantially (Yoo et al., 2019). In this highly connected and continuously updated digital environment, information about popular products, limited releases, countdown reminders, and user-generated display posts is constantly amplified, placing consumers more often in situations where they may feel that they are about to miss something (Dinh & Lee, 2022). At the same time, in an increasingly competitive market, firms have relied on strategies such as scarcity appeals, viral diffusion, and social display to attract consumer attention and stimulate immediate, unplanned purchases (Barton et al., 2022). In this context, Fear of Missing Out (FoMO) helps explain why consumers make rapid purchase decisions when they fear missing a product, an experience, or an opportunity for group identification, and why they remain involved with a brand after purchase (Morsi et al., 2025).

This phenomenon is particularly salient in designer toy consumption, especially in blind box purchase settings (Lee et al., 2025). As an important segment of the cultural and creative industries, designer toys have emerged as a product category that elicits emotion-driven purchasing because of their symbolic meaning, emotional value, and collectible nature (Audretsch & Belitski, 2021). In recent years, with the convergence of technology and cultural creativity, designer toy brands such as Funko, Pop Mart, and Medicom Toy have expanded rapidly and developed growing global market influence (Ericsson et al., 2024). Among them, Pop Mart has become a representative firm in China’s designer toy market (POP Mart, 2025). Pop Mart primarily targets Generation Z consumers with strong purchasing potential and meets their demand for emotional investment in cultural products and for collectibility (Sun et al., 2023). More importantly, the uncertainty and scarcity embedded in its blind box products bring together scarcity cues, social comparison, identity expression, and emotional investment within the same consumption setting, which continues to drive Impulsive Buying (IB) among young consumers (Phan & Hoai, 2025). Compared with general product consumption, designer toy consumption, particularly blind box consumption, combines uncertainty, scarcity, community interaction, and series collection in a more concentrated form (Tang & Zhang, 2026; Wei & Yu, 2025; Whyke et al., 2023). This context therefore provides a suitable setting for examining the formation of FoMO and its behavioral consequences.

Although research on designer toy consumption has grown in recent years, the existing literature has remained centered on product mechanisms and market phenomena, with primary attention given to uncertainty marketing, scarcity and premium effects, and the characteristics of young consumer groups (Lee et al., 2025; Xia et al., 2025; Zhang & Luo, 2024). In contrast, limited attention has been given to the emotional mechanisms underlying such consumption behavior. In the broader consumer behavior literature, Materialism, Envy, and Scarcity have each been shown to shape purchase tendencies and behavioral responses (Jin & Muqaddam, 2021; Rasmussen et al., 2022; Van Tran et al., 2023). These features render blind-box consumption a theoretically distinctive context, as FoMO in this setting is not confined to product acquisition but also involves the loss of symbolic value, social participation, and identity expression. Accordingly, this context provides an appropriate setting for examining the formation of FoMO and its behavioral consequences.

FoMO is associated not only with immediate purchase behavior but also with continued consumer involvement after purchase (Kao & Huang, 2024; Morsi et al., 2025). Prior research has shown that FoMO heightens consumer attention to trends, exclusive experiences, and mainstream brands, and induces responses such as unplanned buying and compulsive buying (Kang & Ma, 2020; Lu & Sinha, 2024; Roberts & David, 2020; Suresh & Biswas, 2020). In addition, the significance of designer toy consumption does not end with a single transaction, but often extends to continued attention, series collection, and Repurchase Intention (RI) (Lee et al., 2025; Xu et al., 2025). Against this background, examining whether FoMO further influences RI through IB is contextually important and can deepen understanding of sustained consumption processes (Morsi et al., 2025; Mustikasari et al., 2025).

Against this backdrop, this study integrates Social Comparison Theory, Self-Determination Theory, and the Stimulus–Organism–Response (S-O-R) framework to examine how Materialism, Envy, and Scarcity influence FoMO, and how FoMO further affects IB, which in turn influences RI. Social Comparison Theory provides the basis for explaining Envy and its behavioral motivation. Self-Determination Theory helps explain FoMO as a negative emotion arising from unmet psychological needs. The S-O-R framework provides the overarching analytical logic for linking antecedent variables, emotional states, and behavioral outcomes.

This study makes three main contributions. First, it brings FoMO into the context of designer toy consumption and addresses the limitation of prior research, which has focused primarily on product mechanisms and market phenomena, by shifting attention to consumer psychological mechanisms. Second, it incorporates Materialism, Envy, and Scarcity into a unified analytical framework and thereby offers a more systematic account of the antecedents of FoMO, deepening understanding of how this specific consumption emotion is formed. Third, it verifies a serial mediation mechanism in which antecedent variables influence RI through FoMO and IB. This finding not only shows the mediating role of FoMO in designer toy consumption, but also shows how FoMO and IB jointly shape subsequent RI. In doing so, the study extends the analytical focus from immediate purchase responses to the post-purchase stage and provides a more complete explanation of sustained behavior in designer toy consumption.

## 2. Literature Review and Hypothesis Development

### 2.1. Materialism

Materialism is commonly defined as a value orientation that emphasizes the importance of acquiring, possessing, and displaying material wealth, which is often regarded as a major source of happiness, success, and social status (Dittmar & Isham, 2022; Richins & Dawson, 1992). In consumption contexts, materialistic consumers are more likely to evaluate themselves and others in terms of the quantity and quality of possessions and are therefore more susceptible to external symbols, social recognition, and identity expression (D. Wu et al., 2021). Compared with products that serve only utilitarian functions, they tend to prefer goods with symbolic meaning and display value and are more responsive to persuasive marketing cues (Huaman-Ramirez & Merunka, 2021; Mehmood et al., 2025). Accordingly, Materialism has been widely used to explain consumers’ pursuit of high-value products such as luxury goods, as well as the formation of purchase intention in influencer-related contexts (Bahri-Ammari et al., 2020; Dinh & Lee, 2022). In trend-driven consumption contexts, this strong emphasis on possession, display, and social recognition may increase concern about failing to obtain desirable products, thereby providing an important psychological basis for the formation of FoMO.

### 2.2. Social Comparison Theory

Social Comparison Theory (Festinger, 1954) proposes that individuals evaluate their own condition by comparing themselves with others in order to reduce cognitive uncertainty and satisfy the need for self-evaluation (Buunk & Gibbons, 2007). In consumption contexts, such comparison is reflected not only in judgments of ability, status, and lifestyle, but also in attention to product ownership, consumption experiences, and identity expression (Jani & Han, 2013). In highly visual social media environments, consumers are more likely to engage in upward comparison through others’ conspicuous consumption, which may produce psychological discrepancy and emotional fluctuation. Prior research has applied Social Comparison Theory to explain behaviors such as sustainable consumption, luxury preference, compulsive buying, and compensatory consumption (Pahlevan et al., 2021; Pillai & Nair, 2021; Usmani & Ejaz, 2020). These findings indicate that the theory has substantial explanatory value for understanding emotional and behavioral responses in consumption contexts.

From this perspective, Envy is one of the most common emotions generated by upward comparison in consumer settings. Envy typically arises when individuals recognize that others possess superior qualities, achievements, or possessions that they themselves lack, and it is accompanied by a desire to obtain those advantages (Parrott & Smith, 1993). Compared with malicious envy, benign envy is more likely to motivate individuals to reduce the gap through effort or action rather than hostility toward others (Jabutay et al., 2025). In consumption contexts, this emotion often heightens sensitivity to the products, experiences, or consumption opportunities possessed by others, thereby strengthening the perception that one may be missing a valuable opportunity.

### 2.3. Scarcity

Scarcity cues are a common and effective marketing tool. Firms often create a sense that an opportunity is fleeting by emphasizing limited product quantity, constrained supply, or strong demand, thereby heightening consumers’ sense of urgency and prompting faster purchase decisions (Phan & Hoai, 2025). In social media marketing contexts, Scarcity often appears in the form of limited-time offers, limited releases, and countdown reminders, and it exerts a significant influence on consumer decision-making and participation (Ho et al., 2024; Suttikun & Mahasuweerachai, 2026). At the same time, Scarcity is not merely an individual-level stimulus. In interactive and socially embedded consumption environments, consumers may perceive limited opportunities more strongly because of others’ simultaneous attention and competition, which creates a collective sense of urgency (Shao et al., 2025). In such contexts, Scarcity may function as a direct situational trigger that intensifies concern about missing a consumption opportunity.

### 2.4. Self-Determination Theory

SDT emphasizes autonomous motivation and the basic psychological needs that underlie individual behavior (Ryan & Deci, 2000). According to this theory, autonomy, competence, and relatedness are basic needs that support psychological well-being and positive behavior. When these needs are satisfied, individuals are more likely to experience well-being, achievement, and intrinsic motivation. When these needs are obstructed, negative emotions such as stress and anxiety are more likely to emerge (Kowal & Fortier, 1999). Therefore, unmet basic psychological needs are often regarded as an important psychological basis for negative emotions.

Within this theoretical framework, FoMO can be understood as a negative emotional experience associated with frustrated psychological needs. In its original meaning, FoMO refers to individuals’ concern that others may be having rewarding experiences from which they are absent (Przybylski et al., 2013). Prior research has shown that lower satisfaction of basic psychological needs is associated with higher levels of FoMO, and that this relationship may be influenced by factors such as relative deprivation (Przybylski et al., 2013; Xie et al., 2018). Thus, FoMO is not merely a general emotional response to “missing out.” It also reflects perceived frustration of relatedness, competence, or autonomy needs. In contexts characterized by social comparison and community interaction, FoMO is closely associated with belongingness, social connection, peer recognition, and identity validation (Roberts & David, 2020).

In consumer and marketing contexts, FoMO has been used to explain consumers’ responses to trends, opportunities, and socially embedded consumption experiences (Good & Hyman, 2020). This consumption-specific nature is more evident in blind-box consumption. Prior studies have shown that blind-box consumption involves not only product purchase, but also series collection, community interaction, and psychological motivation (Wei & Yu, 2025; Xu et al., 2025). Therefore, FoMO in this study is not treated as simple purchase anxiety or immediate urgency induced by scarcity cues, but as a consumption-specific psychosocial mechanism in blind-box consumption. Based on SDT, FoMO is regarded as an emotional state associated with frustrated basic psychological needs and is used in the proposed model to link materialism, envy, and perceived scarcity with IB and RI.

**Figure 1:**
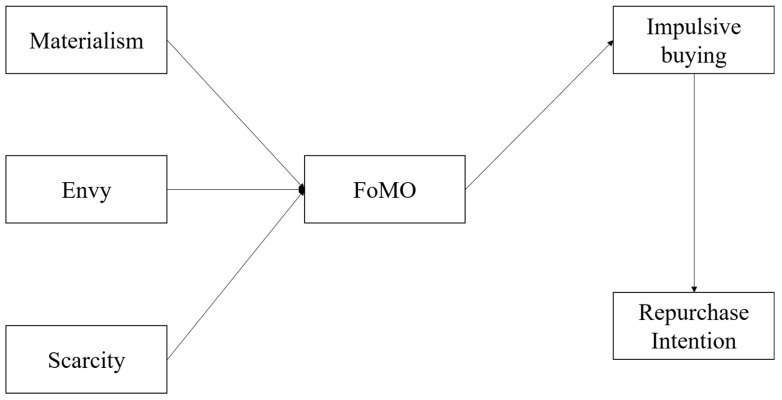
Theoretical model.

Based on the above theoretical discussion, the proposed research model is presented in Figure 1.

### 2.5. Hypothesis Development

#### 2.5.1. Antecedents of FoMO

Because materialistic consumers place greater value on the social recognition, status symbolism, and display value associated with products, they tend to be more sensitive to products linked to popularity, Scarcity, and identity-related meaning in consumption contexts (Huaman-Ramirez & Merunka, 2021; D. Wu et al., 2021). At the same time, individuals with a higher level of Materialism are more likely to be influenced by external marketing information and symbolic cues, which strengthens concern about not possessing a desired product or missing a consumption opportunity (Dinh & Lee, 2022; Mehmood et al., 2025). Therefore, a higher level of Materialism is more likely to increase FoMO.

In social media environments, consumers increasingly engage in social comparison through others’ consumption displays, lifestyles, and product ownership, and such comparison makes them more sensitive to whether they are falling behind others (Caliskan et al., 2024). When others possess products, experiences, or status symbols that they have not yet obtained, consumers are more likely to experience Envy. Prior research has shown that Envy increases consumers’ willingness to pay for related products and also raises the likelihood of purchasing similar products (Cui et al., 2021; Lin, 2018). Benign envy, in particular, although it also arises from psychological discrepancy in comparison, is more likely to motivate individuals to reduce the gap through consumption behavior rather than remain confined to negative emotion itself (Crusius & Mussweiler, 2011). Under such conditions, individuals become concerned not only with what they do not currently have, but also with whether they are missing a product, an opportunity, or a symbolic benefit. As a result, FoMO becomes more likely to arise.

In addition to internal cues, external situational cues may also contribute to the formation of FoMO. Prior research has shown that firms often use promotions, Scarcity, and trend appeals to evoke FoMO, thereby stimulating purchase desire and influencing decision-making (Dinh & Lee, 2022). In platform-based marketing environments, scarcity promotions are further amplified in real time through greater visibility, authenticity, and interactivity, which strengthens consumers’ perception that an opportunity may disappear quickly and promotes stronger purchase responses (Qu et al., 2023). In addition, Scarcity may intensify concern that failure to act immediately will result in the loss of an opportunity by triggering anticipated regret, thereby generating a stronger impulse to act (Yuen et al., 2022). Therefore, when consumers perceive a higher level of Scarcity in a product or opportunity, they are more likely to experience FoMO. Accordingly, the following hypotheses are proposed:

**H1.**  *Materialism has a positive effect on FoMO.*

**H2.**  *Envy has a positive effect on FoMO.*

**H3.**  *Scarcity has a positive effect on FoMO.*

#### 2.5.2. FoMO and Impulsive Buying

FoMO, as a form of anxiety and unease arising from the possibility of missing a product or experience, has been shown to significantly influence young consumers’ shopping motivation and impulsive purchase decisions (Bläse et al., 2024). Prior research has indicated that FoMO often triggers unplanned purchase behaviors, including IB, compulsive overbuying, and follow-up consumption of mainstream brands (Kang & Ma, 2020; Lu & Sinha, 2024; Suresh & Biswas, 2020). When consumers experience a strong sense of urgency because they fear missing popular, exclusive, or scarce products, they are more likely to make unplanned purchase decisions in order to satisfy immediate needs and relieve psychological unease (Djamhari et al., 2024; Servidio et al., 2024). Empirical research has also shown that FoMO is positively associated not only with purchase intention but also with behaviors such as compulsive buying and excessive consumption (Hussain et al., 2023). These findings indicate that FoMO does not remain at the level of emotional experience, but can be translated into direct consumption behavior. Accordingly, the following hypothesis is proposed:

**H4.**  *FoMO has a positive effect on IB.*

#### 2.5.3. Impulsive Buying and Repurchase Intention

IB is commonly defined as a sudden, immediate, and unplanned purchase behavior in which consumers make decisions quickly under the force of a strong purchase desire, often accompanied by excitement and hedonic appeal (Gupta et al., 2021; Rook, 1987). This behavior reflects the pursuit of immediate gratification and a temporary weakening of self-regulation in a specific context (Rook & Fisher, 1995). Prior research has shown that, in social media environments, consumers are more likely to engage in weakly controlled shopping behavior in response to external information cues (Kumar & Kumar, 2024). In particular, when a product carries strong hedonic attributes, symbolic meaning, or emotional value, the likelihood of IB further increases (Xia et al., 2025).

The post-purchase consequences of IB may differ across consumption contexts. IB can be associated with positive evaluations when the purchase provides enjoyment, satisfaction, or a valued consumption experience (Goel et al., 2022; Silalahi et al., 2025). However, it may also be associated with post-purchase regret, cognitive dissonance, or lower satisfaction when the purchase is later perceived as unnecessary or inconsistent with consumers’ expectations (Beikverdi et al., 2024; Hou, 2025).

In consumption contexts characterized by emotional value, experiential attributes, and sustained attention, IB does not necessarily end with a single transaction, but may serve as the starting point for subsequent RI. Before purchase, consumers typically form expectations regarding product design, quality, and overall experience. When the actual experience meets or exceeds these expectations, satisfaction increases, which in turn strengthens RI (Wang et al., 2023). Prior research has also shown that, in marketing contexts, external stimuli can further enhance RI by promoting IB (Tan et al., 2024). Therefore, although IB appears as an immediate purchase response, it may also translate into stronger RI in contexts characterized by sustained consumption. Accordingly, the following hypothesis is proposed:

**H5.**  *IB has a positive effect on RI.*

Further, FoMO and IB may serve as sequential mediators in the proposed model. Prior research has shown that FoMO is associated with consumers’ tendency toward IB and related consumption behavior (Djafarova & Bowes, 2021). At the same time, FoMO has been found to be closely associated with continued participation behavior and to mediate the relationship between user expectations and continued usage intention, suggesting that FoMO is related to immediate purchase responses and subsequent sustained behavior (Fioravanti et al., 2021). In addition, related research has shown that affective variables and FoMO, when treated as mediating mechanisms, help explain the relationship between emotional states and behavioral responses within the S-O-R framework (Yue et al., 2025). Accordingly, this study proposes a serial mediation model in which FoMO and IB serve as mediators between the antecedent variables and RI. The following hypotheses are therefore proposed:

**H6.**  *FoMO and IB have a serial mediating effect on the relationship between Materialism and RI.*

**H7.**  *FoMO and IB have a serial mediating effect on the relationship between Envy and RI.*

**H8.**  *FoMO and IB have a serial mediating effect on the relationship between Scarcity and RI.*

## 3. Methodology

### 3.1. Measures

All core constructs in this study were measured using five-point Likert scales ranging from 1 (“strongly disagree”) to 5 (“strongly agree”). The measurement items (Table A1) were adapted from established scales in prior studies and were revised to fit the consumption context of cultural and creative products.

IB was measured using a three-item scale developed by Lazim et al. (2020), which captures consumers’ sudden and difficult-to-control purchase impulses in response to external stimuli. FoMO was measured using three items adapted from Przybylski et al. (2013). The items were adjusted to the blind-box consumption context to reflect consumers’ concerns about missing relevant consumption opportunities. Because blind-box consumption often involves limited-edition products and uncertain availability, the contextualized FoMO items included references to limited products. These references were used to situate FoMO in the blind-box consumption context, rather than to measure perceived Scarcity itself. RI was measured using a three-item scale adopted from Li et al. (2025) to assess consumers’ tendency to continue purchasing from the brand in the future. Materialism was measured using the classic scale developed by Richins and Dawson (1992), which reflects the importance consumers attach to material possessions and consumption values. Envy was measured using items adapted from the scale proposed by Lange and Crusius (2015) and reflects consumers’ emotional and behavioral responses when others possess new products. Scarcity was measured using the instrument developed by W.-Y. Wu et al. (2012), which captures consumers’ subjective perception of product scarcity and competitive purchasing situations. Thus, Scarcity was treated as a separate antecedent variable, whereas FoMO was measured as a consumption-specific emotional response to the possibility of missing out.

Before the formal distribution of the questionnaire, the measurement items were reviewed for content validity by two professors and one doctoral researcher. Based on their suggestions, the wording of the questionnaire and the applicability of the items were refined to ensure measurement accuracy and contextual fit.

### 3.2. Data Collection and Sample Characteristics

The questionnaire was administered through Sojump, a widely used online survey platform in China. Respondents who completed the survey received platform points that could be redeemed for cash. To ensure sample relevance, all respondents were required to be familiar with the Pop Mart brand and its major products and to have purchased at least one Pop Mart product. Data were collected between November and December 2025. This period did not coincide with any peak in Pop Mart’s market popularity, which helped reduce the risk of sample bias associated with short-term trends or highly salient events. At the beginning of the questionnaire, respondents were informed that all answers should be based on actual consumption experience. The survey was completed anonymously, and respondents were told that their answers would be used only for academic research and that their personal information would be kept confidential. In addition, screening questions and logic-check items were included to exclude respondents who did not meet the research criteria or provided invalid responses, thereby helping ensure data quality and the reliability of the findings. A total of 518 valid questionnaires were retained for subsequent reliability and validity assessment and empirical analysis.

As shown in Table 1, the demographic characteristics of the sample were summarized across four dimensions: gender, age, education, and monthly income. Female respondents accounted for 54.2% of the sample, exceeding the proportion of male respondents. In terms of age, the 23–27 group accounted for the largest share, followed by the 18–23 group, whereas the 28–32 group and the over-32 group accounted for smaller shares, indicating a generally young sample profile. In terms of education, respondents with a bachelor’s degree or above accounted for a relatively large proportion, including 61.4% with a bachelor’s degree and 11.8% with a master’s degree or above, whereas those with a junior college education or below accounted for 26.8%. In terms of income, the largest group consisted of respondents with a monthly income of RMB 3001–6000, followed by those earning RMB 3000 or below, whereas the shares of respondents earning RMB 6001–8000, RMB 8001–10,000, and above RMB 10,000 were smaller. Overall, the sample was dominated by young and highly educated respondents, and its profile closely matched the core consumer group for blind box products. Taken together, the sample showed both concentration and variation in gender, age, education, and income, providing a sound demographic basis for the subsequent analysis.

## 4. Results

### 4.1. Measurement Model

SPSS 26 was first used to assess the suitability of the data for factor analysis. The overall Kaiser–Meyer–Olkin (KMO) value was 0.864, exceeding the recommended threshold of 0.80, which indicates that the data were suitable for further analysis. Because the data were collected using self-reported measures, common method bias (CMB) was assessed (Podsakoff et al., 2024). Harman’s single-factor test showed that the cumulative variance explained by a single factor was 35.889%, below the recommended threshold of 50% (Podsakoff et al., 2003). In addition, the PLS algorithm in SmartPLS 4 was applied using the default maximum of 300 iterations. As shown in Table 2, all variance inflation factor (VIF) values were below 3.3 (Kock, 2015). Taken together, these results indicate that CMB was not a serious concern in this study.

Moreover, all factor loadings exceeded 0.70. The Cronbach’s alpha values for all constructs were above 0.70, indicating good internal consistency. The average variance extracted (AVE) values were all above 0.50, supporting convergent validity. In addition, the composite reliability (CR) values for all constructs exceeded 0.70, indicating satisfactory reliability.

Discriminant validity was further assessed using the heterotrait–monotrait ratio of correlations (HTMT). The HTMT values should be significantly below the threshold of 0.85 (Hair et al., 2021). As shown in Table 3, all results met this criterion, indicating adequate discriminant validity.

Discriminant validity was further assessed using the Fornell–Larcker criterion. According to this criterion, the square root of the average variance extracted (AVE) for each construct should exceed its correlations with other constructs. As shown in Table 4, this condition was satisfied in all cases. Specifically, the lowest diagonal value was 0.880, which exceeded the highest inter-construct correlation of 0.538, thereby confirming discriminant validity.

### 4.2. Explanatory Power and Model Fit

The explanatory power of the structural model was assessed. The results showed that the R^2^ values for FoMO (R^2^ = 0.353), IB (R^2^ = 0.284), and RI (R^2^ = 0.289) indicated acceptable explanatory power. As shown in Table 5, the effect size (f^2^) results indicated that FoMO and IB made substantial contributions to the explanation of IB and RI, respectively, whereas Envy, Materialism, and Scarcity also contributed to the explanation of FoMO. The Q^2^ values were further examined. The Q^2^ values for FoMO, IB, and RI were 0.260, 0.217, and 0.194, respectively, all of which were above zero, thereby confirming the predictive relevance of the model. Finally, model fit was assessed. The standardized root mean square residual (SRMR) value was 0.057, which met the recommended criterion, indicating that the model fit the data well and was suitable for subsequent analysis.

### 4.3. Structural Model Evaluation

**Figure 2:**
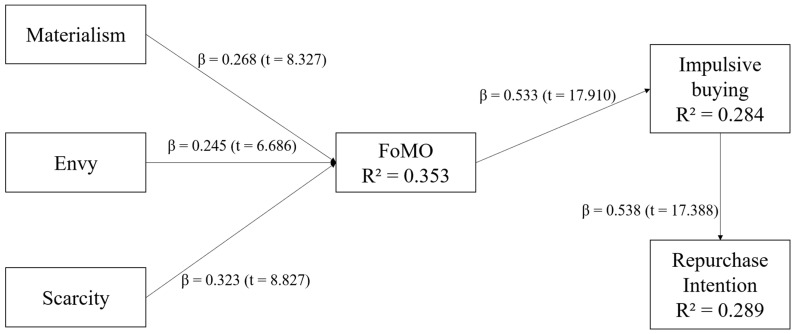
Structural model results.

The structural model was evaluated using SmartPLS with bootstrapping based on 5000 resamples. The results are reported in Table 6, and the structural model is presented in Figure 2. All path coefficients were significant, with t-values above 1.96 and *p*-values below 0.001. Accordingly, all proposed hypotheses were supported. Specifically, Materialism, Envy, and Scarcity were positively associated with FoMO. FoMO was positively associated with IB, and IB was positively associated with RI. Among the antecedent variables, Scarcity showed the strongest relationship with FoMO. In the subsequent structural paths, FoMO showed a substantial positive relationship with IB, and IB showed a substantial positive relationship with RI.

As shown in Table 7, this study further examined the serial mediating roles of FoMO and IB in the relationships between the antecedent variables and RI. The results indicated that all three serial mediation paths were statistically significant. These findings supported the proposed serial mediation model in which FoMO and IB served as sequential mediators between the antecedent variables and RI. Accordingly, FoMO and IB jointly served as a serial mediation mechanism linking the antecedent variables to RI.

## 5. Discussion and Implications

### 5.1. Discussion

This study examined the proposed model using a sample of Chinese Generation Z consumers. All hypotheses were supported. Overall, the findings indicate that RI in blind-box consumption is associated with both consumer-related factors and market-related cues. These results provide a basis for discussing consumers’ psychological responses and purchase tendencies in designer toy consumption.

First, Materialism was positively associated with FoMO. This result can be understood in relation to the view that Materialism emphasizes the connection between possession, happiness, and social status (Górnik-Durose, 2020). Materialism also shapes consumer motivation and value judgment (Cavazos-Arroyo & Puente-Díaz, 2026). In the designer toy context, products often carry symbolic, collectible, and identity-related meanings. Consumers with a high level of Materialism may therefore interpret failure to obtain popular or meaningful products as a loss of self-expression or social comparison advantage. This suggests that FoMO is related not only to market cues, but also to consumers’ value orientation toward possession. For brands, product stories, design meanings, and collectible value may help strengthen consumer interest more effectively than purely functional product communication.

Second, Envy was positively associated with FoMO, highlighting the role of social comparison in this context. This study further extends comparative emotion into the formation process of FoMO (Tandon et al., 2024). The Envy measured in this study refers to benign envy, which reflects a catch-up-oriented psychological state activated by others’ possession status (Shahzad et al., 2026). In blind-box consumption, display posts, unboxing content, early possession, and social sharing make ownership more visible and create comparison points among consumers. As a result, envy may be associated with stronger concern about falling behind others or being excluded from shared consumption experiences. This implies that community interaction and user-generated content can enhance engagement, but such communication should encourage shared participation rather than direct competitive pressure.

Third, Scarcity was positively associated with FoMO and showed the strongest relationship among the antecedent variables. This finding is consistent with the view that Scarcity increases consumers’ sensitivity to limited opportunities (Abdrabbo et al., 2025). In blind-box consumption, scarcity is embedded in limited releases, hidden editions, uncertain availability, and collection completion. Therefore, missing a scarce item may mean more than failing to obtain a product; it may also imply losing a collectible, symbolic, or socially visible opportunity. This helps explain why FoMO in this context may be closely related to identity signaling and social belonging. Limited releases may increase consumer interest, but their effectiveness depends on meaningful product design, collection logic, and community experience rather than pressure-based promotion alone.

Fourth, FoMO was positively associated with IB, and IB was positively associated with RI. This finding suggests that FoMO may reduce consumers’ willingness to delay purchase decisions in blind-box consumption. When consumers perceive that a product may soon become unavailable or socially less accessible, immediate purchase may serve as a response to perceived loss. The result is consistent with prior studies showing that FoMO strengthens impulsive and unplanned purchasing (Djamhari et al., 2024; Servidio et al., 2024). It is also consistent with research showing that IB further promotes RI (Tan et al., 2024). These findings suggest that FoMO may reduce the space for rational evaluation and make immediate decision-making more likely. Because RI reflects the likelihood of future purchase, this result suggests that an initial impulse purchase may be associated with continued purchase intention when consumers evaluate the consumption experience positively. For brands, the post-purchase experience remains important, because continued purchase intention is more likely when consumers evaluate the purchase experience positively.

Finally, the results supported the serial mediating roles of FoMO and IB in the relationships between the antecedent variables and RI. Consistent with prior research suggesting that FoMO is associated with impulsive consumption responses and that IB may be related to sustained consumption tendencies (Djafarova & Bowes, 2021; Fioravanti et al., 2021), this finding suggests that FoMO and IB should be considered together when explaining RI in designer toy consumption. Within the proposed model, FoMO reflects the emotional response associated with Materialism, Envy, and Scarcity, whereas IB represents the purchase response through which this emotional response is further related to RI. From a practical perspective, brands should avoid relying only on one-time stimulation. Greater attention should be paid to whether the post-purchase experience supports positive evaluation and continued purchase intention.

### 5.2. Theoretical Implications

The theoretical contributions of this study are reflected in three main aspects. First, this study contributes to the literature by conceptualizing blind-box consumption as a distinctive context for examining consumption-specific FoMO. Rather than treating blind-box consumption as a simple empirical application, this study shows that uncertainty, scarcity, collection, and community visibility are combined in this setting. This perspective shifts attention from product mechanisms and market phenomena to consumers’ psychological responses in designer toy consumption.

Second, this study contributes to the literature by identifying different sources of FoMO in blind-box consumption. By incorporating Materialism, Envy, and Scarcity into one analytical framework, the study shows that FoMO is associated with both consumer-related factors and market-related cues. In particular, the finding that Scarcity showed the strongest relationship with FoMO suggests that contextual market cues deserve greater attention in explaining consumption-specific FoMO. This contribution clarifies the roles of value orientation, social comparison, and perceived market opportunity as distinct antecedent sources of FoMO.

Third, this study provides empirical support for the serial mediating roles of FoMO and IB in the relationship between consumer and market antecedents and RI. Rather than treating FoMO as an isolated emotional variable, the study positions FoMO and IB as related mechanisms within the proposed model. This contribution refines the application of the S-O-R framework in designer toy consumption by showing how consumer predispositions and market cues are associated with emotional and behavioral responses.

### 5.3. Practical Implications

This study also offers several practical implications for designer toy brands, cultural and creative enterprises, and related marketing practice. First, brands should recognize that FoMO is not an abstract form of anxiety that arises spontaneously, but a context-dependent emotional response associated with specific consumption cues. The findings show that Scarcity is the strongest antecedent of FoMO, suggesting that limited-quantity and limited-time release strategies may increase consumers’ sensitivity to missed opportunities. For brands, such strategies may be useful in contexts such as new product launches, co-branded releases, holiday-limited editions, and hidden-edition releases, especially when they are supported by clear product value and collectible appeal.

Second, brands should pay close attention to differences in consumers’ underlying psychological orientations when designing marketing strategies. The findings indicate that Materialism and Envy were also positively associated with FoMO, suggesting that consumers are drawn to designer toys in part because they associate these products with identity expression, social comparison, and self-worth. In practice, this means that brands may emphasize the symbolic value, subcultural meaning, and display value of products in their communications in order to increase consumer psychological involvement. At the same time, community interaction, user-generated posts, unboxing content, and social sharing may enhance product attractiveness by making consumption experiences more visible and participatory.

Third, brands should treat IB as a possible basis for subsequent RI rather than simply as the endpoint of a one-time transaction. The findings show that, in the context of designer toy and blind box consumption, immediate purchases formed under emotional influence may be related to sustained purchasing. For brands, this means that immediate purchase responses can be encouraged through limited releases, social display, trend diffusion, and subcultural interaction. However, such purchases should also be supported by product quality, unboxing experience, collection value, intellectual property extension, and community management, so that an initial impulse may be associated with stronger RI under favorable consumption experiences. Brands therefore should not view IB and RI as separate outcomes. Instead, they should continue to strengthen positive consumer experience and brand connection after the initial purchase.

### 5.4. Limitations and Future Research

This study has several limitations that also point to directions for future research. First, the sample was drawn mainly from Chinese Generation Z consumers with prior experience purchasing Pop Mart products, and the research context was limited to designer toy consumption. As consumer responses may differ across cultural backgrounds, consumption environments, and product categories, future research could extend the present model to other cultural and creative products or to cross-cultural contexts in order to further examine its robustness. Second, this study relied on cross-sectional data, which limits its ability to examine changes in FoMO, IB, and RI over time. Future research could adopt longitudinal designs, experimental methods, or tracking surveys to examine the temporal relationships among these variables more precisely. Third, the outcome variable in this study was limited to RI. In addition, this study focused on RI as a positive post-purchase outcome and did not examine possible negative outcomes of IB, such as regret, cognitive dissonance, or lower satisfaction. Future research could examine actual repurchase behavior and other post-purchase outcomes. Incorporating actual purchase behavior, brand loyalty, community participation, and user sharing behavior into the analytical framework would provide a more comprehensive understanding of the influence of FoMO on consumers’ long-term behavioral outcomes. Fourth, constructs such as FoMO and Materialism are conceptually broad. Future research could use more comprehensive scales to capture their multidimensional nature.

## Figures and Tables

**Table 1:** Demographic Profile of Respondents.

Particulars	Description	Values	%
Gender	Male	237	45.8
	Female	281	54.2
Age (years)	18–23	145	28.0
	24–27	228	44.0
	28–32	73	14.1
	Above 32	72	13.9
Education Level	Associate degree or below	139	26.8
	Bachelor’s degree	318	61.4
	Master’s degree/PhD	61	11.8
Income (monthly) (RMB)	Below 3000	150	29.0
	3001–6000	231	44.6
	6001–8000	68	13.1
	8001–10,000	47	9.1
	Above 10,000	22	4.2

**Table 2:** Results of reliability and validity.

Construct	Loadings	VIF	Cronbach’s Alpha	CR	AVE
Materialism			0.850	0.908	0.768
Materialism1	0.876	2.424			
Materialism2	0.880	1.972			
Materialism3	0.872	2.016			
Envy			0.760	0.862	0.676
Envy1	0.835	1.678			
Envy2	0.822	1.630			
Envy3	0.808	1.404			
Scarcity			0.814	0.890	0.729
Scarcity1	0.852	1.809			
Scarcity2	0.876	1.963			
Scarcity3	0.833	1.679			
FoMO			0.830	0.898	0.746
FoMO1	0.846	1.755			
FoMO2	0.874	2.030			
FoMO3	0.872	1.981			
Impulsive Buying (IB)			0.854	0.911	0.774
IB1	0.872	2.088			
IB2	0.885	2.105			
IB3	0.882	2.139			
Repurchase Intention (RI)			0.784	0.873	0.696
RI1	0.815	1.704			
RI2	0.844	1.567			
RI3	0.844	1.649			

**Table 3:** Discriminant Validity: HTMT Results.

	Envy	FoMO	IB	Materialism	RI	Scarcity
Envy						
FoMO	0.498					
IB	0.463	0.633				
Materialism	0.142	0.437	0.466			
RI	0.299	0.578	0.647	0.317		
Scarcity	0.470	0.582	0.493	0.287	0.425	

**Table 4:** Discriminant validity: Fornell–Larcker Criterion.

	Envy	FoMO	IB	Materialism	RI	Scarcity
Envy	0.822					
FoMO	0.397	0.864				
IB	0.372	0.533	0.880			
Materialism	0.119	0.375	0.404	0.876		
RI	0.231	0.466	0.538	0.271	0.834	
Scarcity	0.371	0.479	0.412	0.242	0.340	0.854

**Table 5:** F^2^ Effect Size Results.

Paths	f-Square
Envy → FoMO	0.080
FoMO → IB	0.397
IB → RI	0.406
Materialism → FoMO	0.105
Scarcity → FoMO	0.132

**Table 6:** Path Coefficients and Hypothesis Testing.

Hypothesis	Paths	Path Factor	Sample Mean	STDEV	*t* Values	*p* Values	Conclusion
H1	Materialism→ FoMO	0.268	0.270	0.032	8.327	0.000 ***	Accepted
H2	Envy → FoMO	0.245	0.246	0.037	6.686	0.000 ***	Accepted
H3	Scarcity → FoMO	0.323	0.323	0.037	8.827	0.000 ***	Accepted
H4	FoMO → IB	0.533	0.534	0.030	17.910	0.000 ***	Accepted
H5	IB → RI	0.538	0.538	0.031	17.388	0.000 ***	Accepted

Note: *** *p* < 0.001.

**Table 7:** Serial Mediation Results.

Hypothesis	Paths	Path Factor	Sample Mean	STDEV	*t* Values	*p* Values
H6	Materialism→FoMO→ IB→RI	0.077	0.078	0.012	6.360	0.000 ***
H7	Envy → FoMO → IB →RI	0.070	0.071	0.013	5.547	0.000 ***
H8	Scarcity → FoMO → IB → RI	0.092	0.093	0.014	6.565	0.000 ***

Note: *** *p* < 0.001.

## Data Availability

The raw data supporting the findings of this study have been uploaded during the submission process for editorial evaluation. Due to confidentiality agreements with the respondents, the dataset is not publicly available. Anonymized raw data, with all respondent-identifying information removed, are available from the corresponding author upon reasonable request.

## Abbreviations

The following abbreviations are used in this manuscript:

IB	Impulsive Buying
FoMO	Fear of Missing Out
S-O-R	Stimulus–Organism–Response
RI	Repurchase Intention

## Appendix A

**Table A1:** Measurement Items.

Construct	
Materialism	
Materialism1	I would be happier if I could afford to buy more things.
Materialism2	My life would be better if I owned certain things I don’t have.
Materialism3	I like to own things that impress people.
Envy	
Envy1	When others have new products, I wish I had them too.
Envy2	When others have new products, I try to find ways to get them.
Envy3	I ask others if they have gotten the new products.
Scarcity	
Scarcity1	I think many people will buy this product.
Scarcity2	I think there is a limited supply of this product right now.
Scarcity3	I think this product will sell out soon.
FoMO	
FoMO1	When I see others post about limited-edition products, I want to buy them too.
FoMO2	I worry that others have more products than I do.
FoMO3	I feel bothered when I miss out on limited-edition products.
Impulsive buying (IB)	
IB1	I buy things immediately without considering if I really need them.
IB2	I often buy unplanned products when shopping.
IB3	When limited-edition products are released, I easily feel the urge to buy them.
Repurchase Intention (RI)	
RI1	I want to buy this product again.
RI2	I would choose this brand again next time.
RI3	I am likely to repurchase this product.
